# Supplementary material for: Diversity of lanternfish (Myctophidae) larvae along the Ninety East Ridge, Indian Ocean
Source: PeerJ. 2025 Mar 17;13:e19144. doi: 10.7717/peerj.19144 (PMC11925047; doi:10.7717/peerj.19144)
Supplement: Supplemental Information 3 [file peerj-13-19144-s003.pdf]

Contents

Supplementary Figure. 1. *Benthosema suborbitale* 2
Supplementary Figure. 2. *Bolinichthys distofax* 3
Supplementary Figure. 3. *Bolinichthys photothorax* 4
Supplementary Figure. 4. *Bolinichthys* sp.1 5
Supplementary Figure. 5. *Ceratoscopelus warmingii* 6
Supplementary Figure. 6. *Dasyscopelus* sp. 7
Supplementary Figure. 7. *Diaphus brachycephalus* 8
Supplementary Figure. 8. *Diaphus effulgens* 9
Supplementary Figure. 9. *Diaphus fragilis* 10
Supplementary Figure. 10. *Diaphus mollis* 11
Supplementary Figure. 11. *Diaphus parri* 12
Supplementary Figure. 12. *Diaphus perspicillatus* 13
Supplementary Figure. 13. *Diaphus phillipsi* 14
Supplementary Figure. 14. *Diaphus richardsoni* 15
Supplementary Figure. 15. *Diaphus* sp.1 16
Supplementary Figure. 16. *Diaphus* sp.2 17
Supplementary Figure. 17. *Diaphus* sp.3 18
Supplementary Figure. 18. *Diaphus* sp.4 19
Supplementary Figure. 19. *Diaphus splendidus* 20
Supplementary Figure. 20. *Diaphus termophilus* 21
Supplementary Figure. 21. *Hygophum* sp. 22
Supplementary Figure. 22. *Lampadena luminosa* 23
Supplementary Figure. 23. *Lampadena* sp.1 24
Supplementary Figure. 24. *Lampadena* sp.2 25
Supplementary Figure. 25. *Lampanyctus nobilis* 26
Supplementary Figure. 26. *Lampanyctus* sp.1 27
Supplementary Figure. 27. *Lampanyctus* sp.2 28
Supplementary Figure. 28. *Lampanyctus* sp.3 29
Supplementary Figure. 29. *Lampanyctus* sp.4 30
Supplementary Figure. 30. *Lampanyctus tenuiformis* 31
Supplementary Figure. 31. Myctophidae incertae sedis 32
Supplementary Figure. 32. *Notolychnus valdiviae* 33
Supplementary Figure. 33. *Symbolophorus rufinus* 34
Supplementary Figure. 34. *Symbolophorus* sp.1 35
Supplementary Figure. 35. *Symbolophorus* sp.2 36
Supplementary Figure. 36. *Triphoturus nigrescens* 37

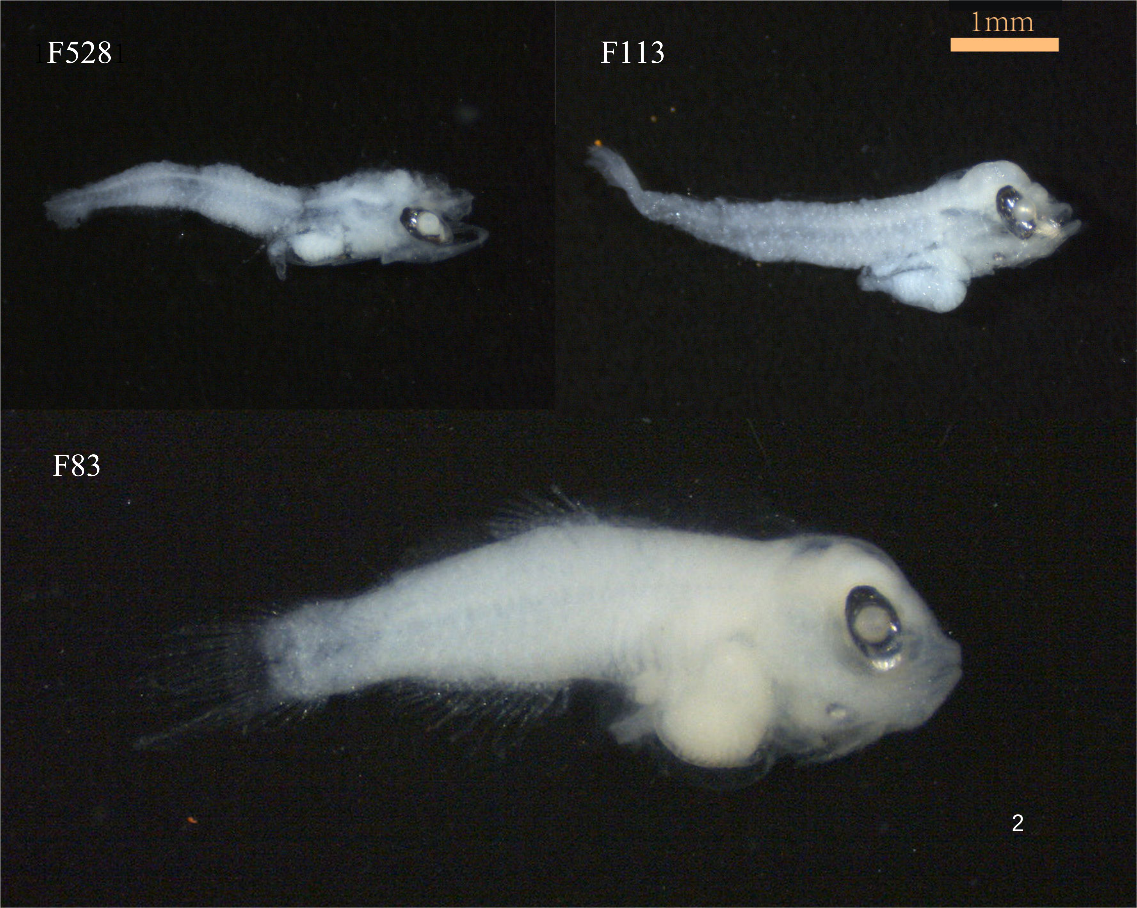
F528
F113
1mm
F83
2

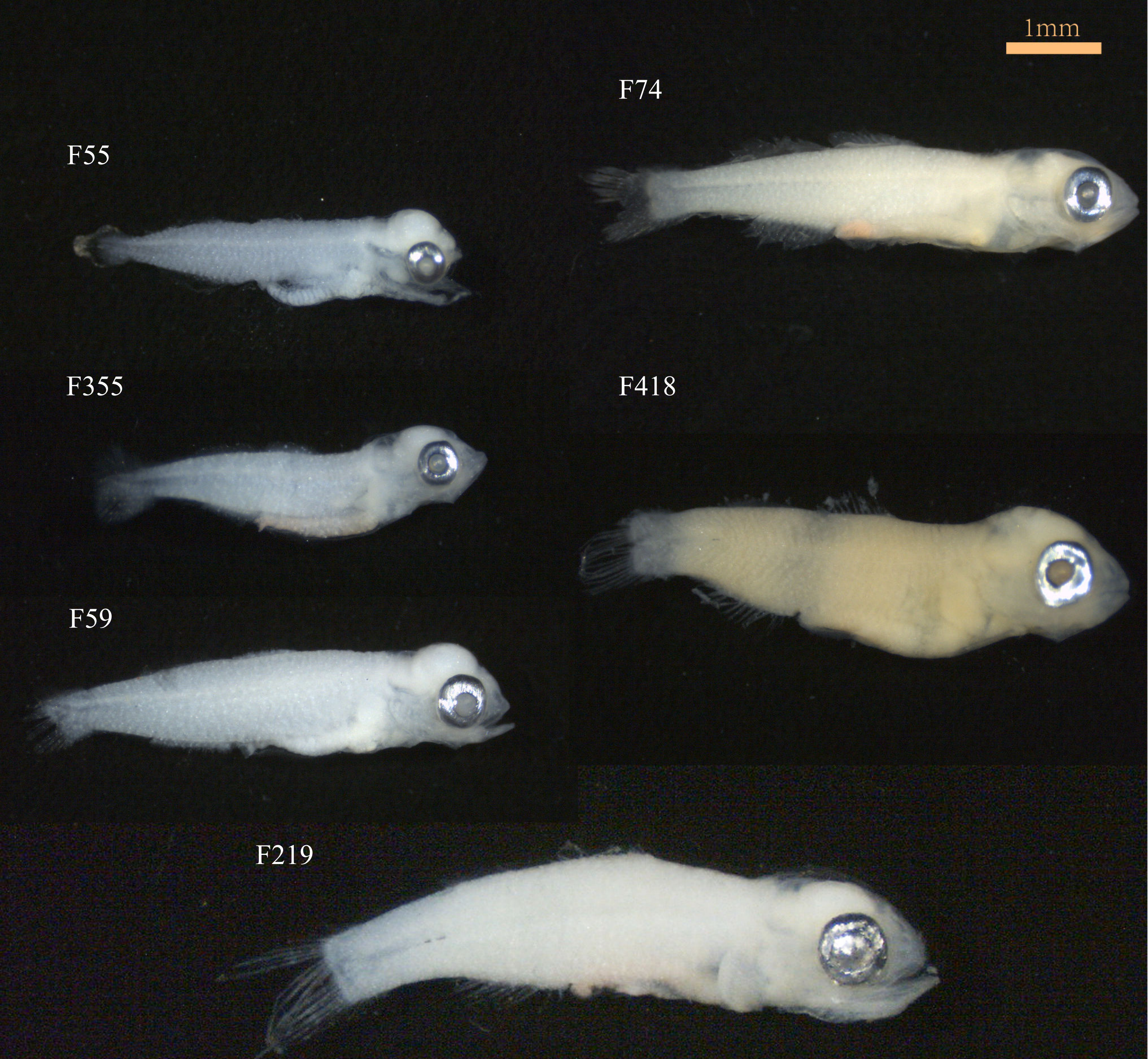
1mm
F74
F55
F355
F418
F59
F219

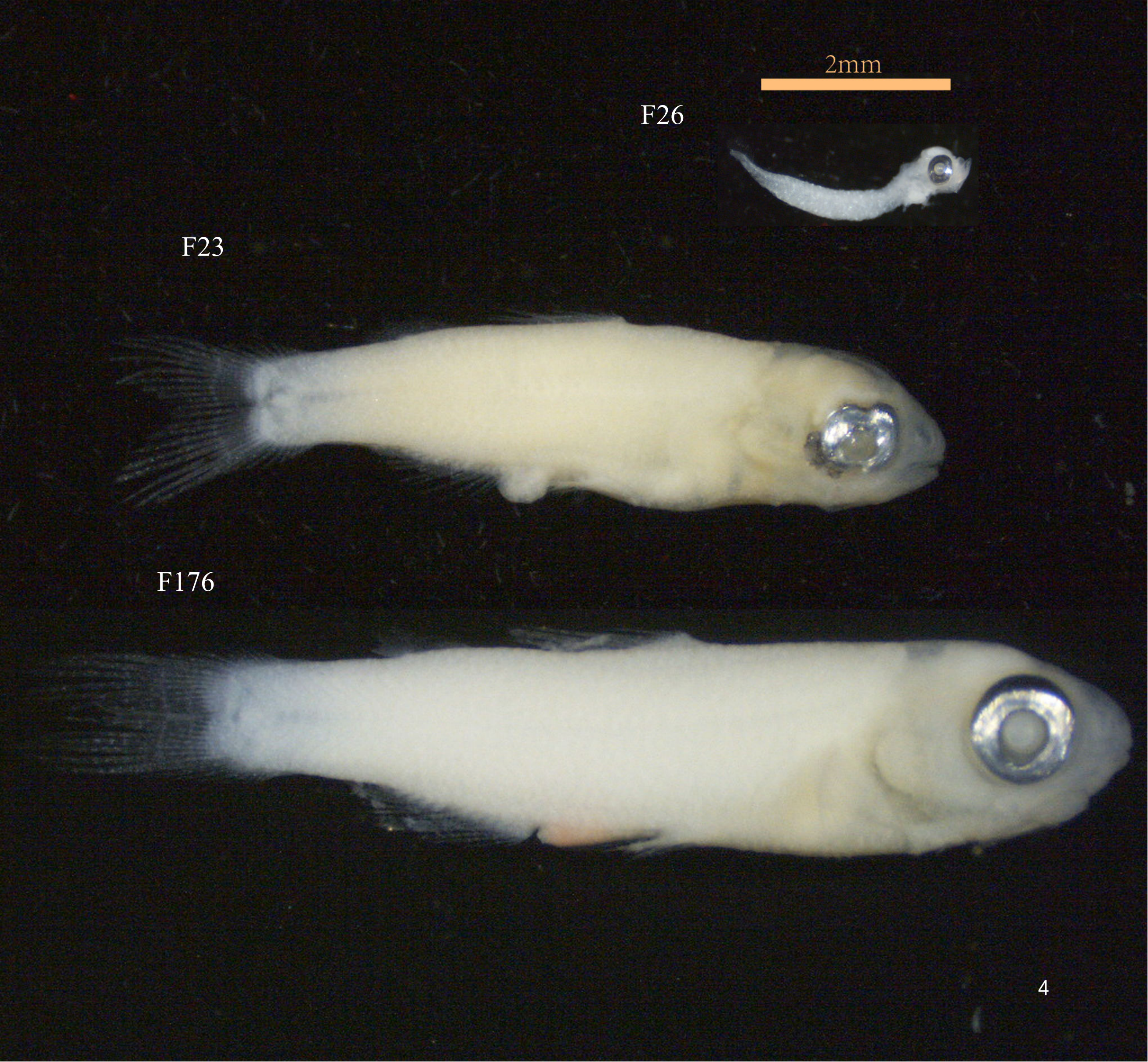
2mm
F26
F23
F176

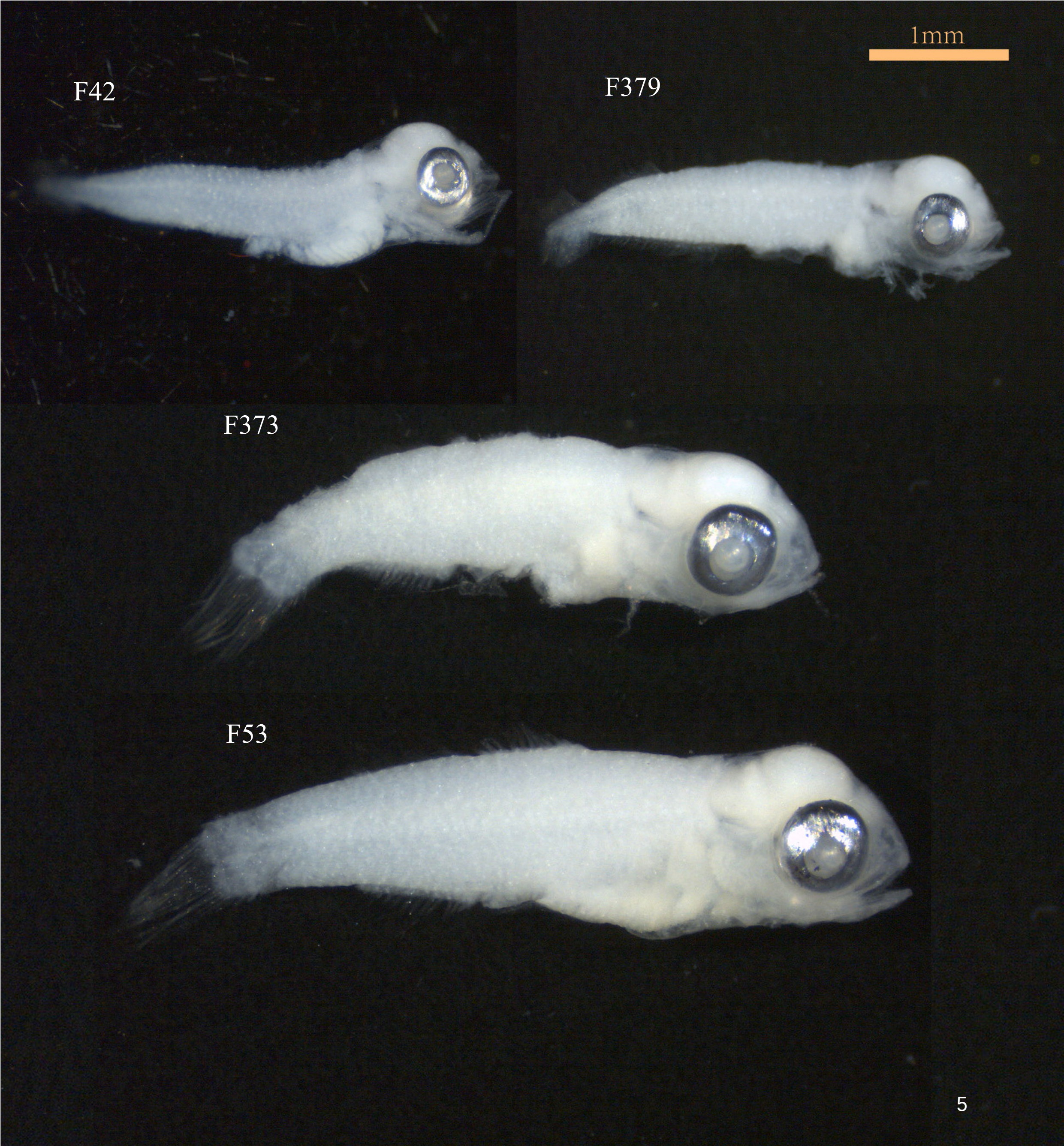
1mm
F42
F379
F373
F53

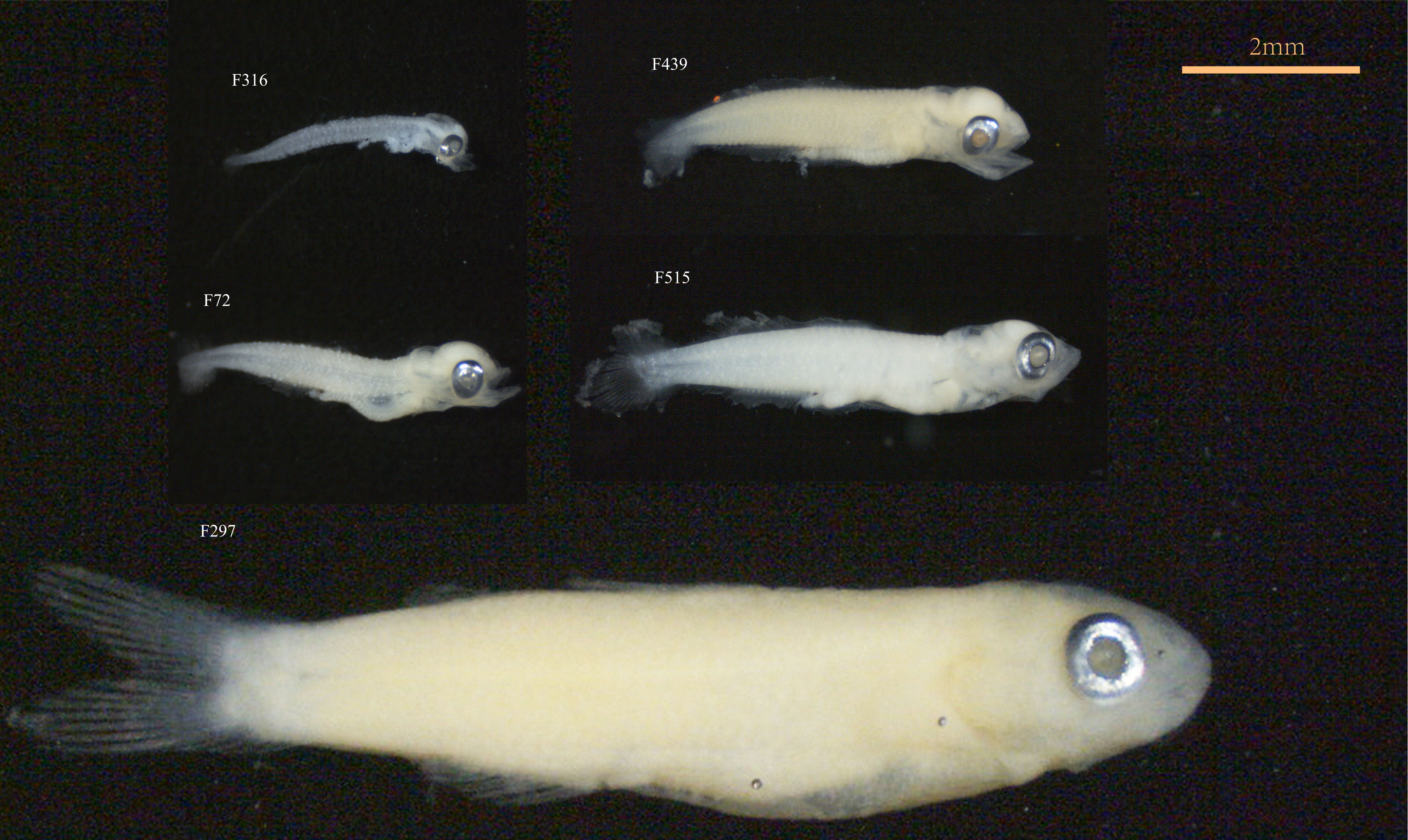
2mm
F316
F439
F72
F515
F297

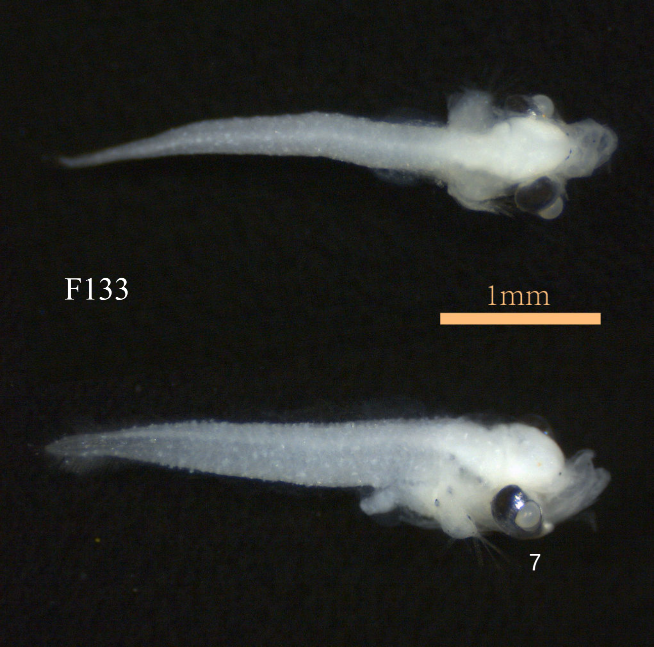
F133
1mm

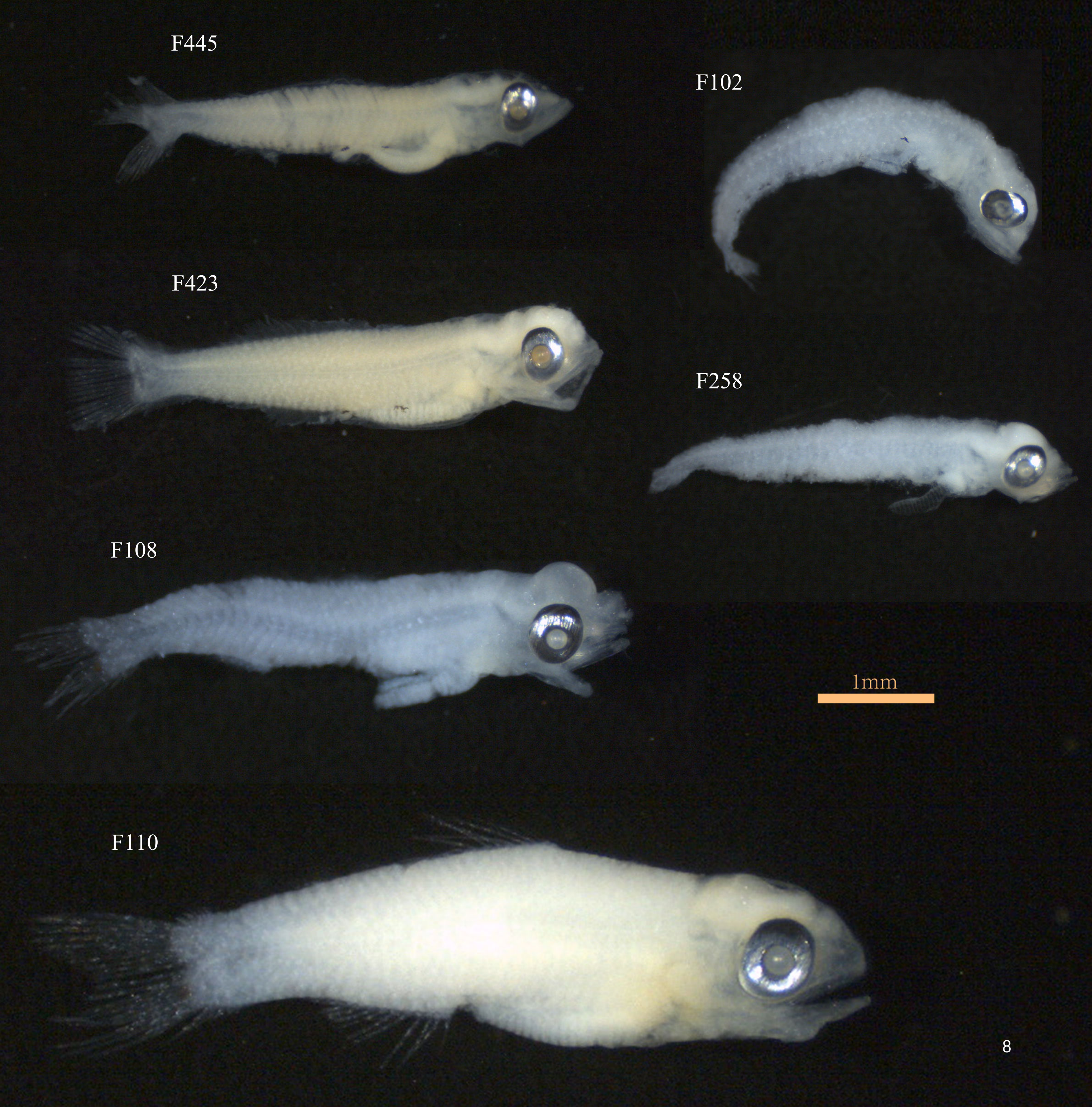
F445
F102
F423
F258
F108
1mm
F110

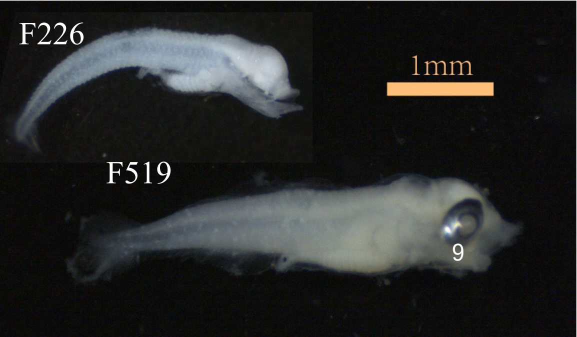
F226
1mm
F519
9

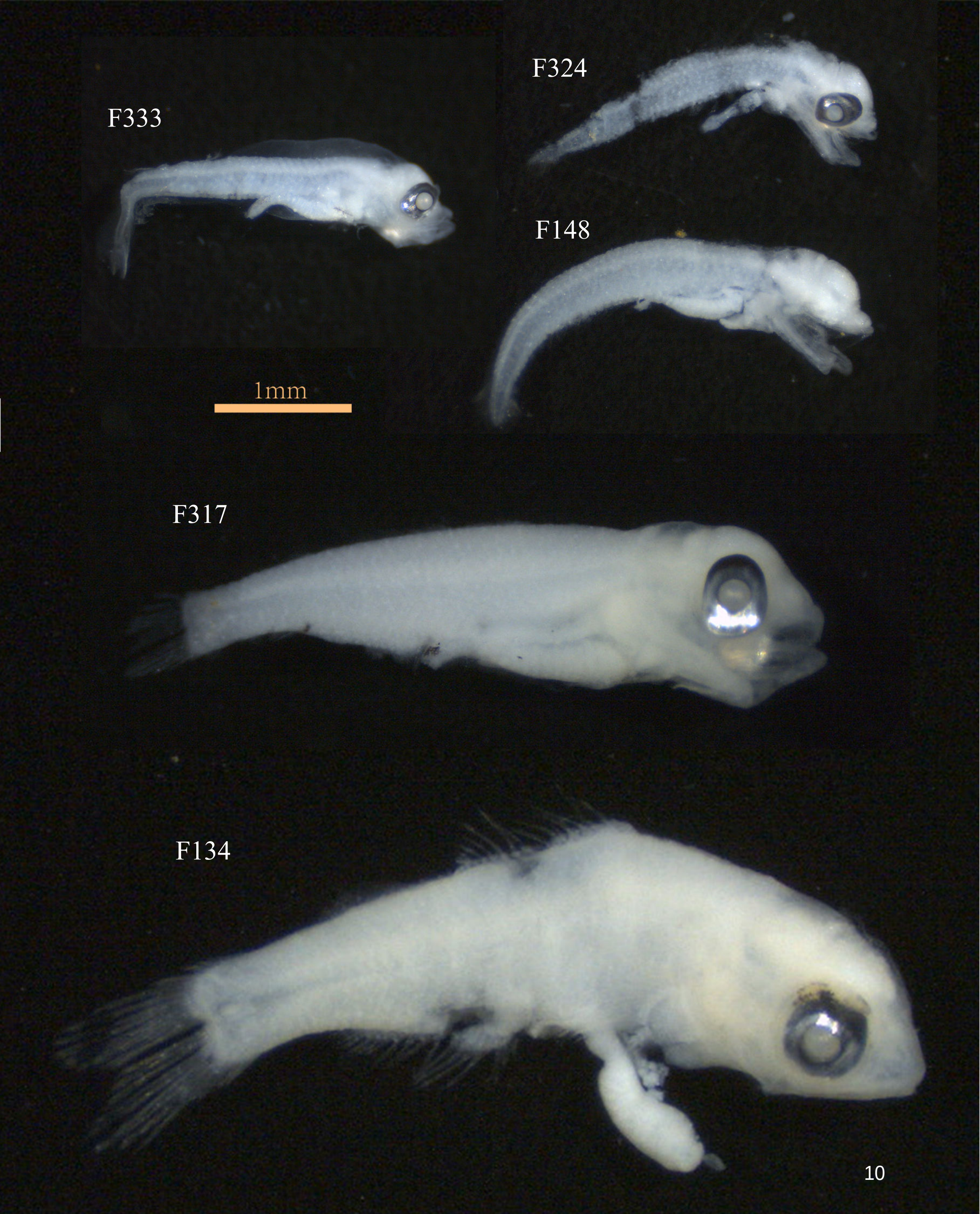
F333
F324
F148
1mm
F317
F134

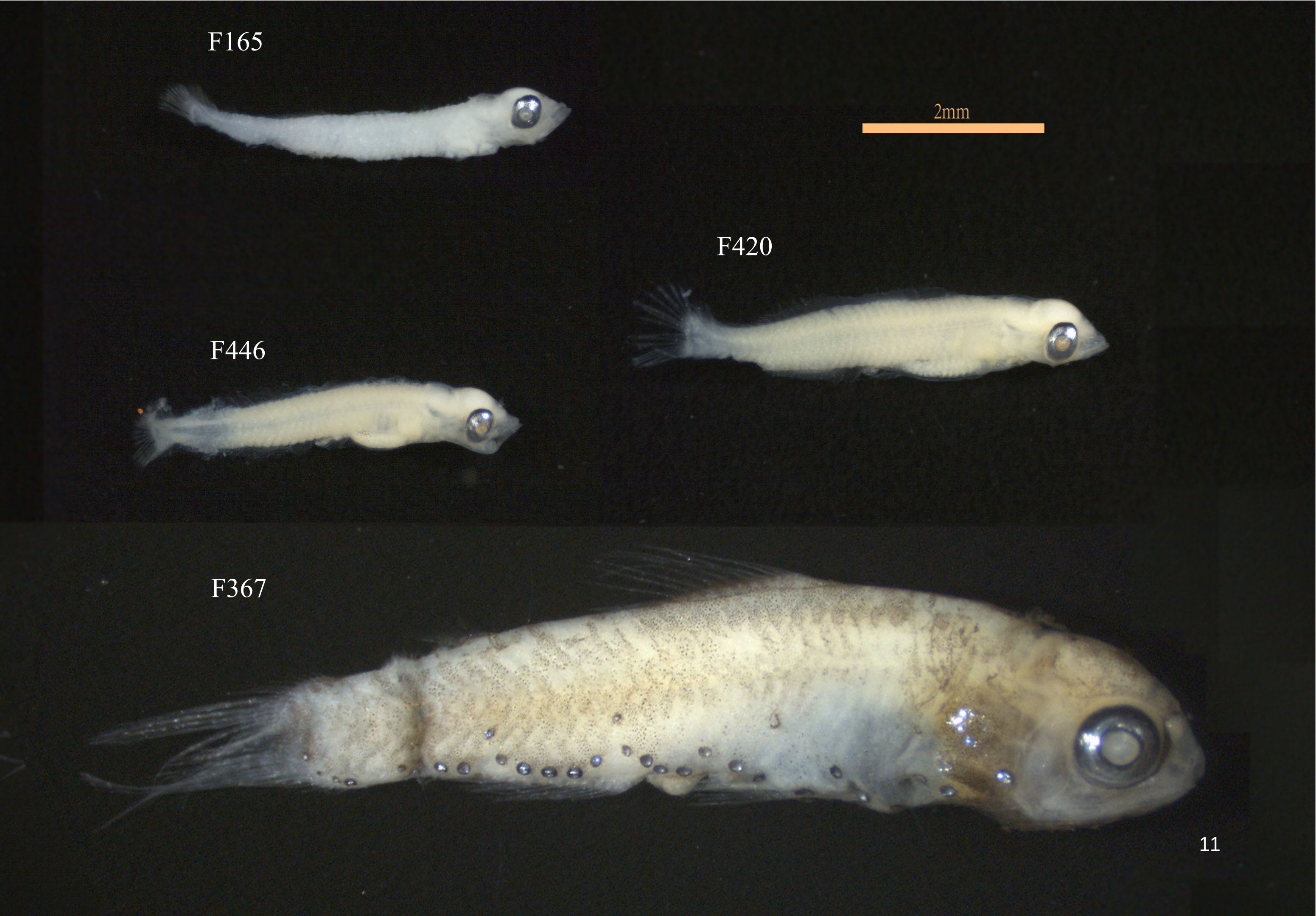
F165
2mm
F420
F446
F367

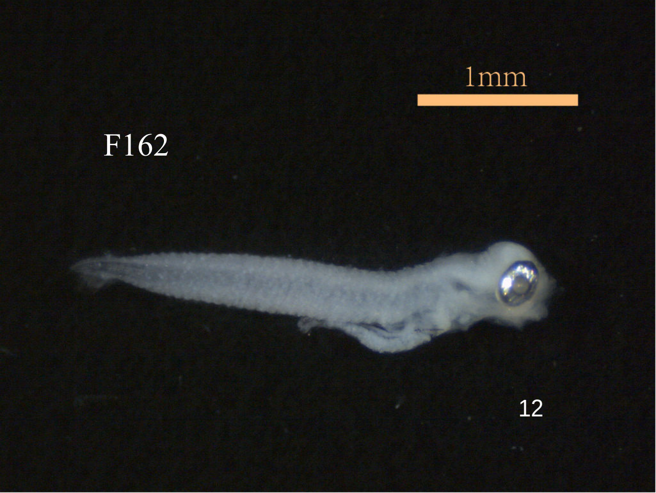
1mm
F162

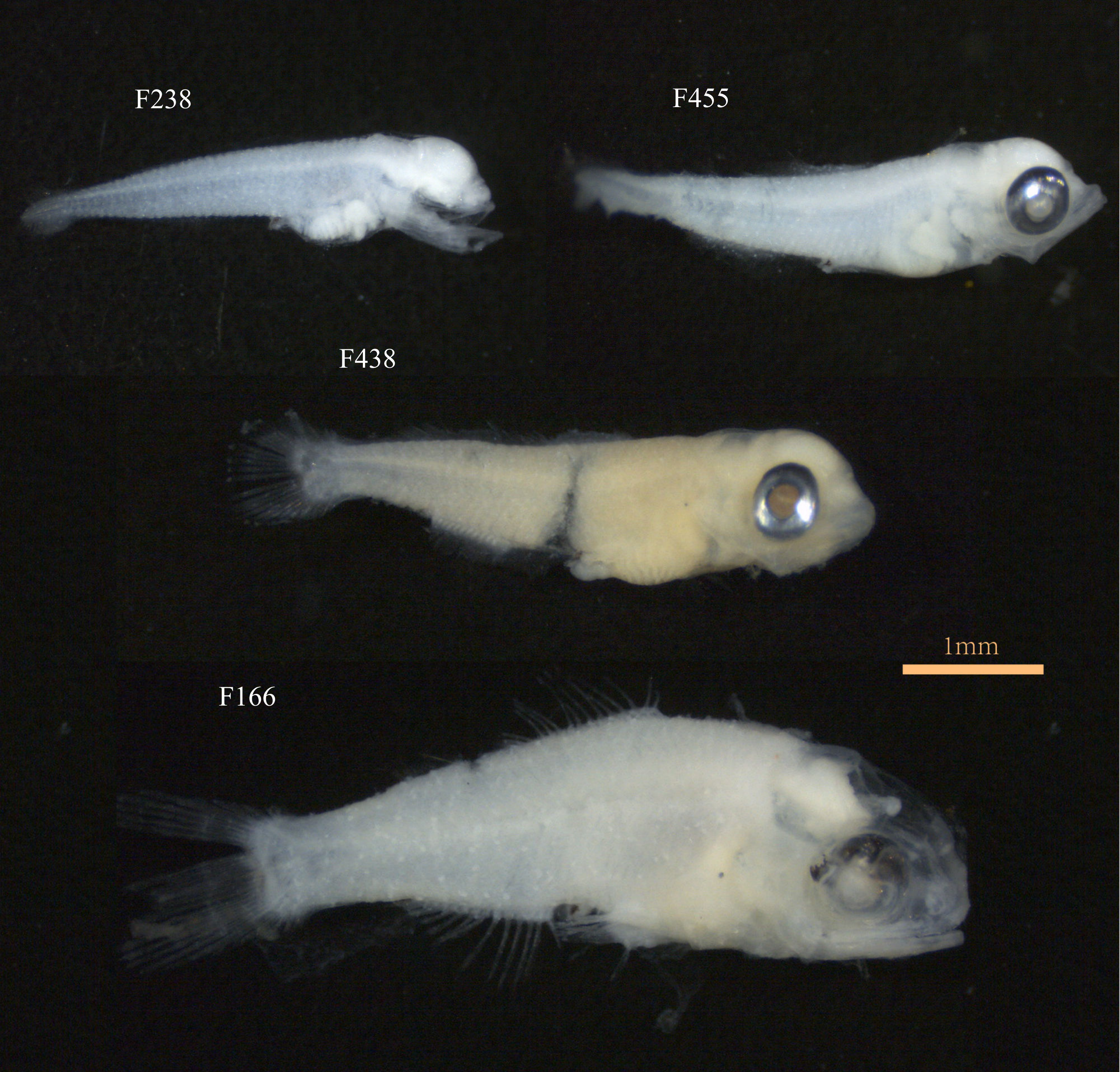
F238
F455
F438
1mm
F166

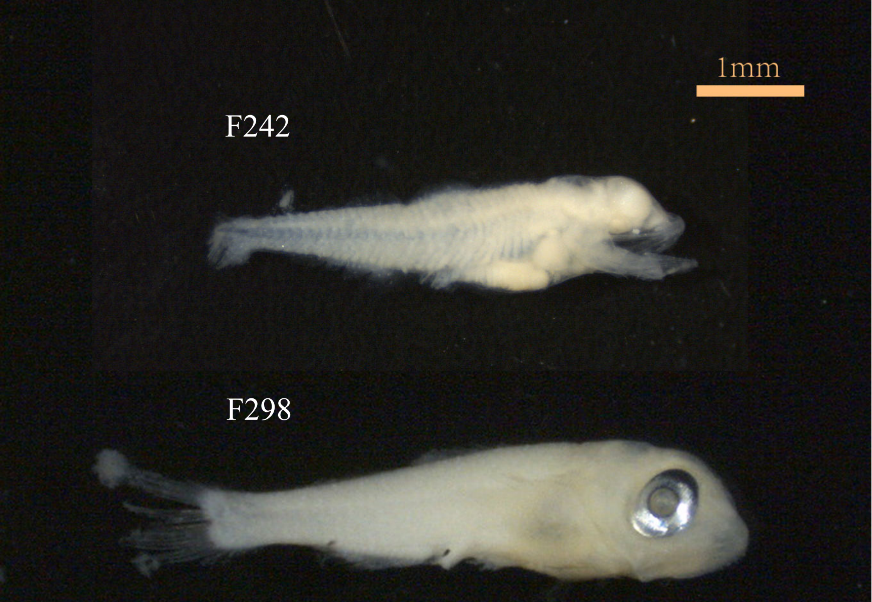
1mm
F242
F298

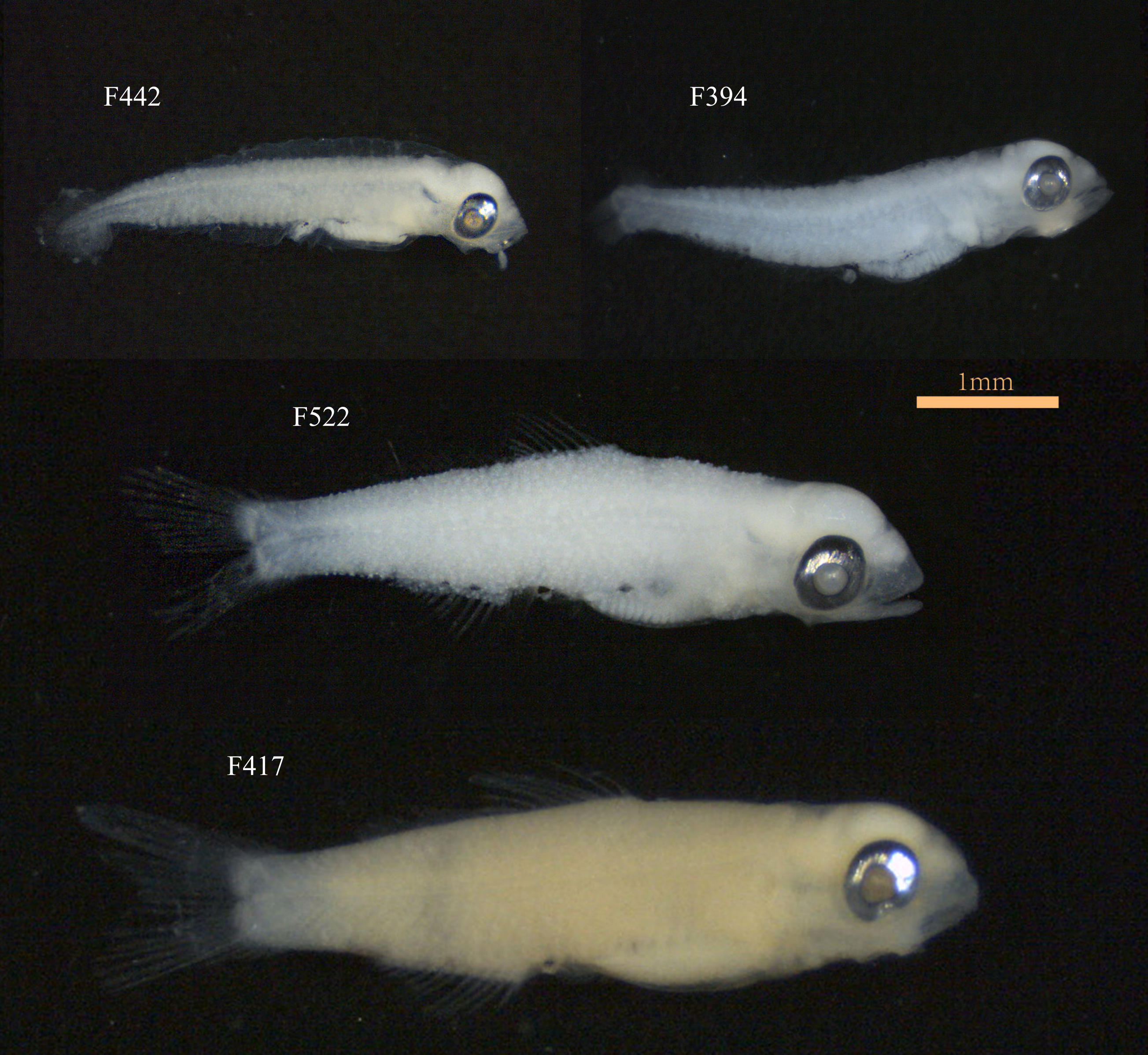
F442
F394
1mm
F522
F417

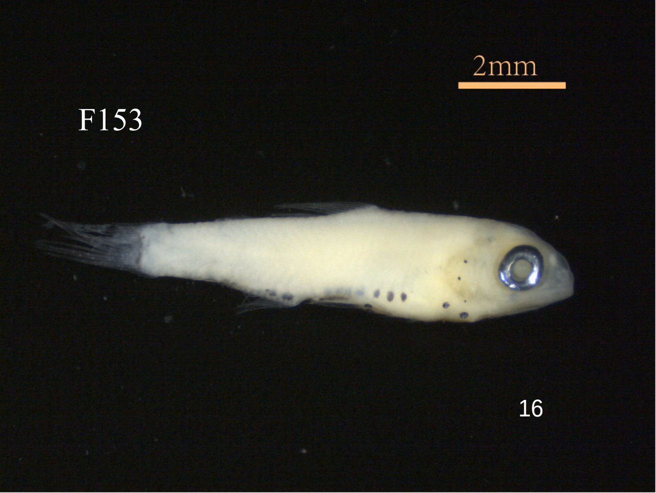
2mm
F153
16

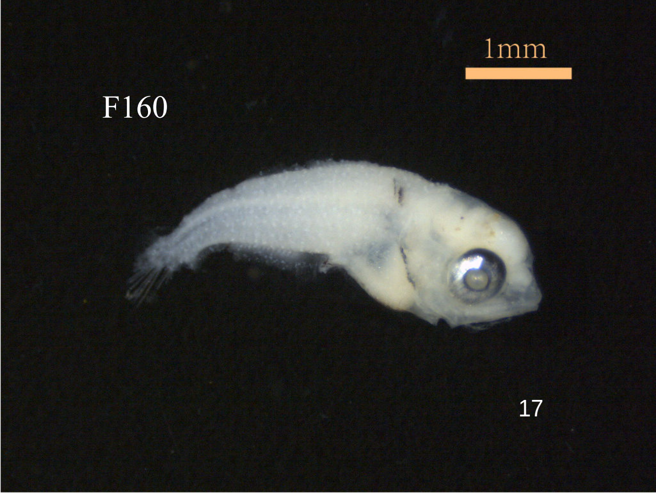
1mm
F160

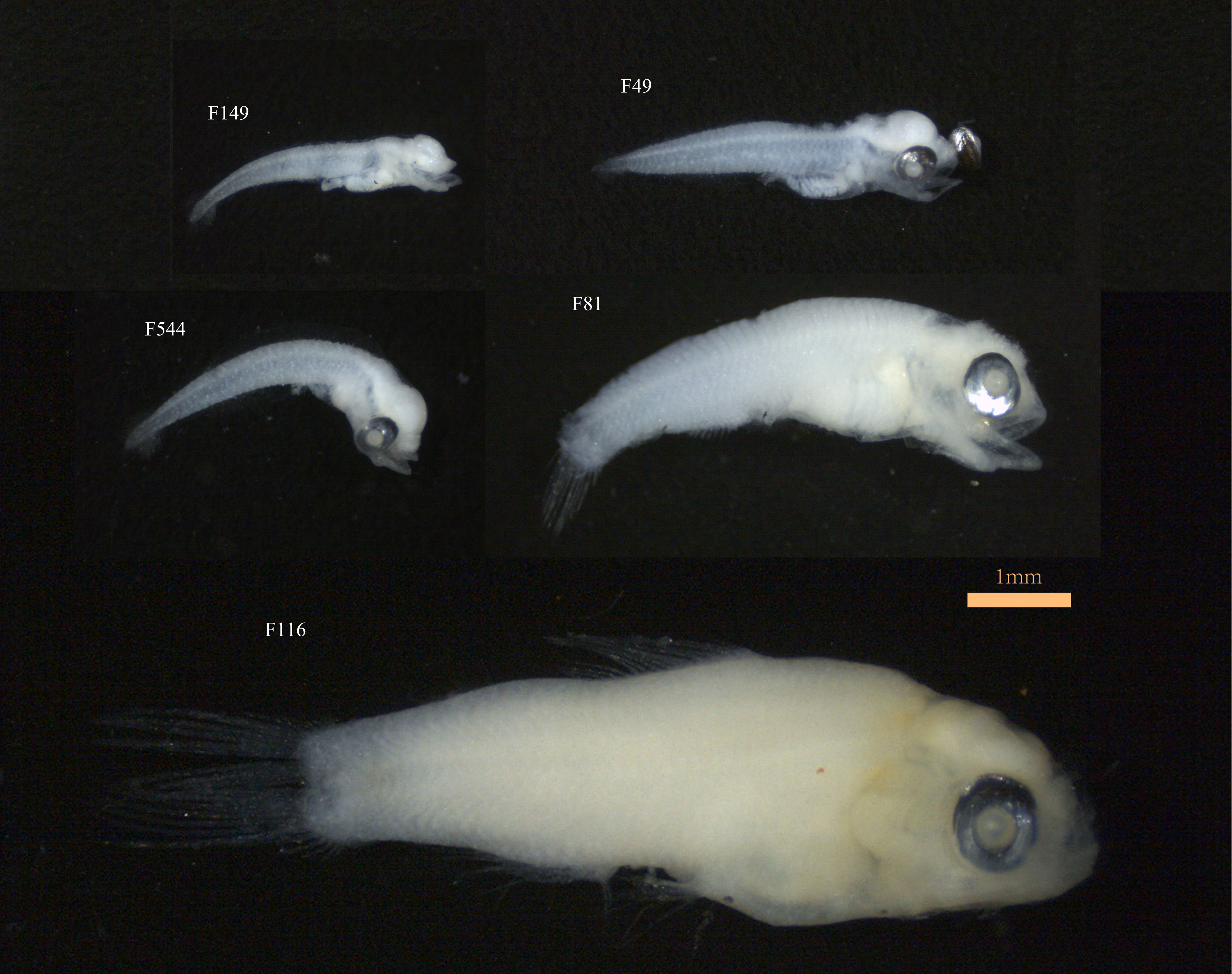
F149
F49
F544
F81
1mm
F116

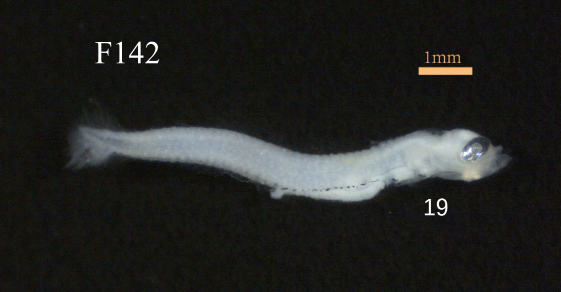
F142
1mm
19

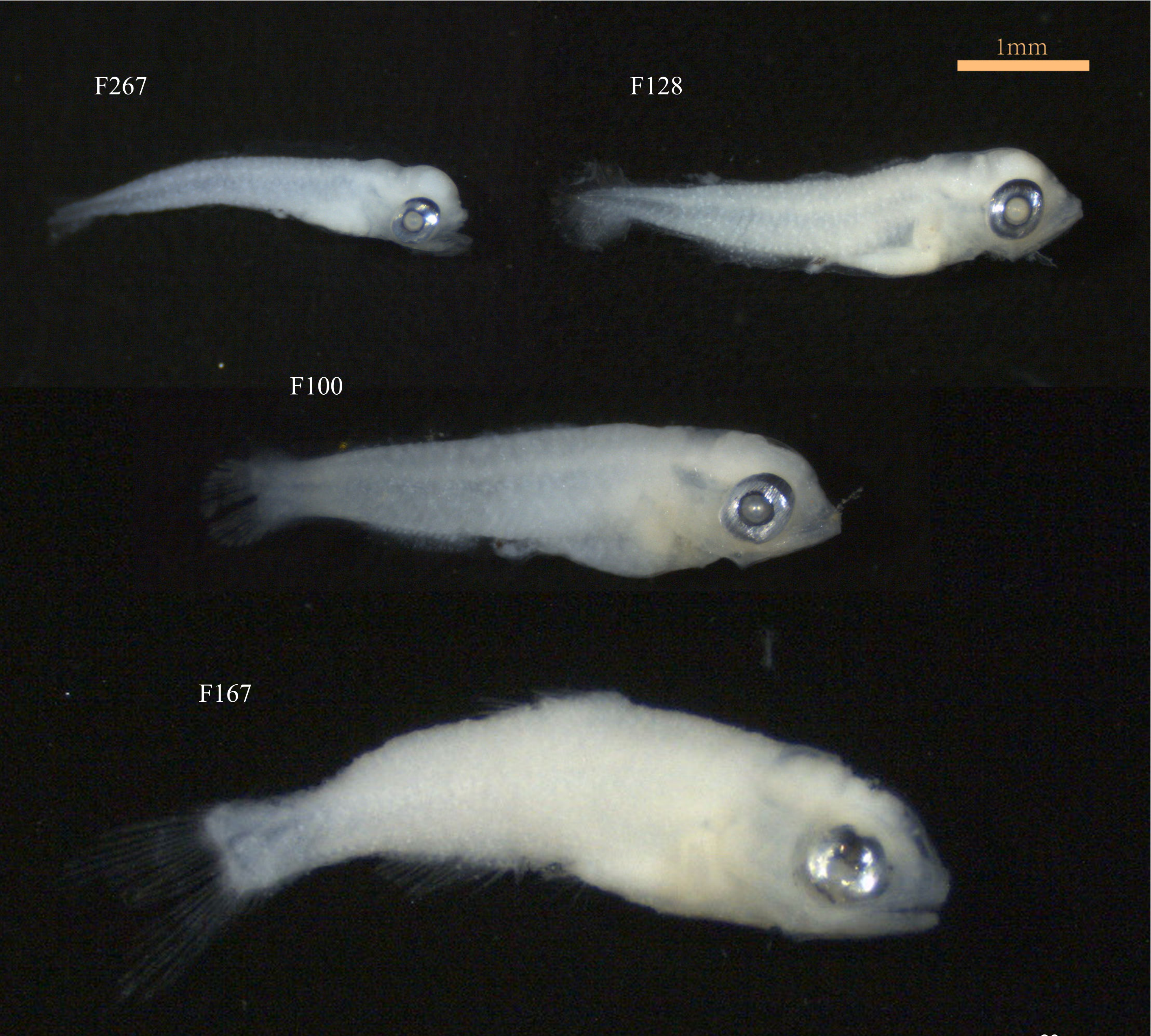
1mm
F267
F128
F100
F167

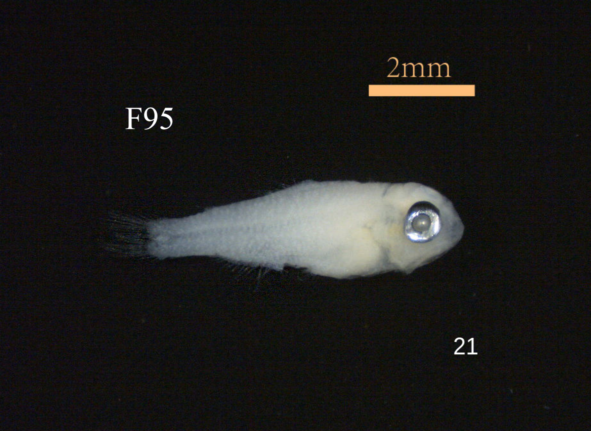
2mm
F95

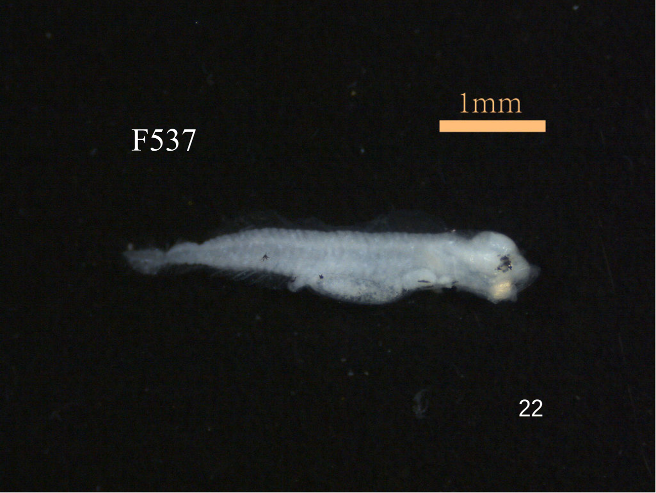
F537
1mm

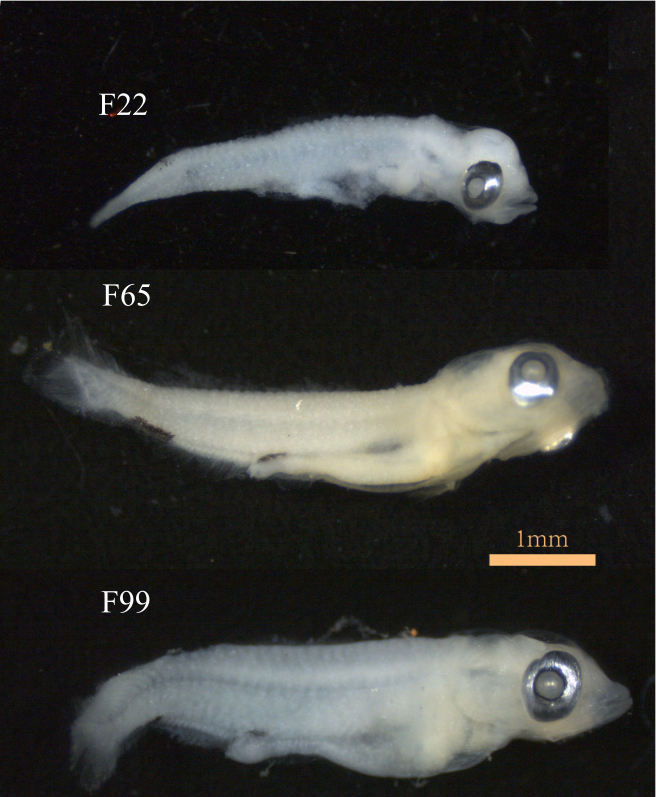
F22
F65
1mm
F99

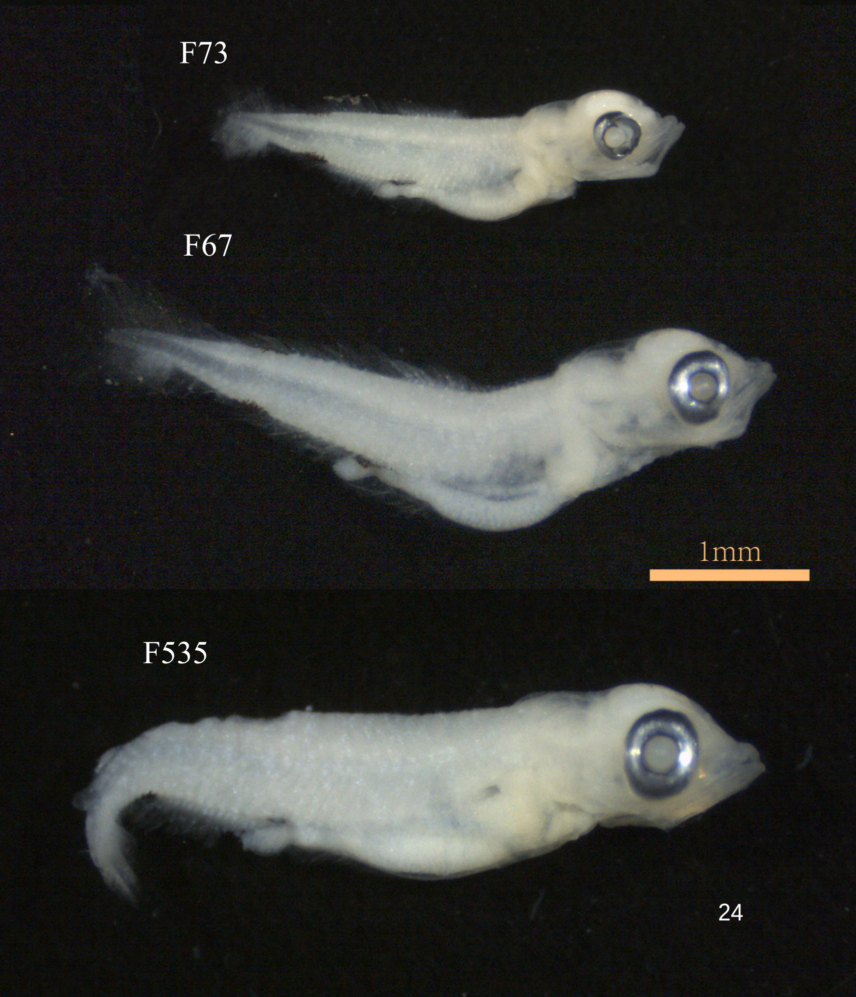
F73
F67
1mm
F535

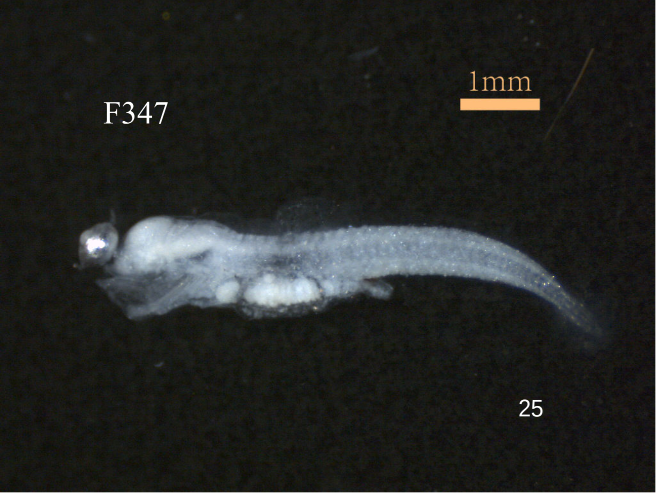
F347
1mm

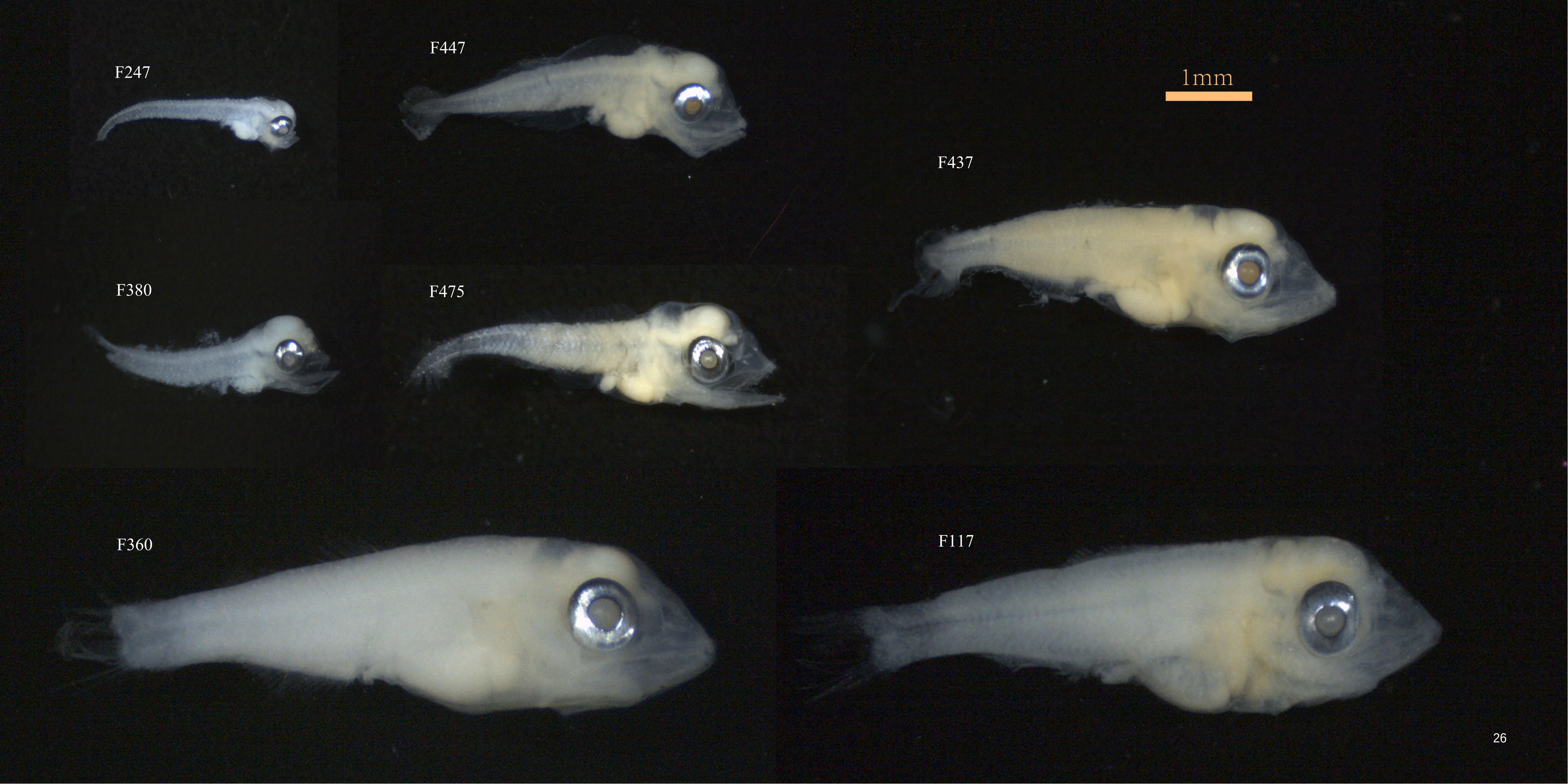
F247
F447
1mm
F437
F380
F475
F360
F117

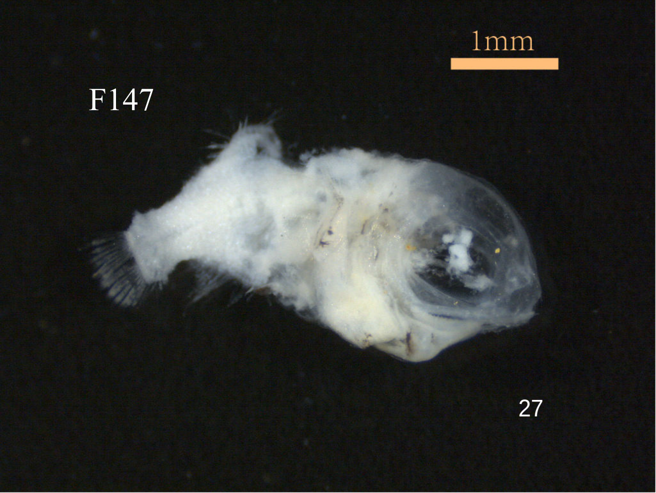
F147
1mm

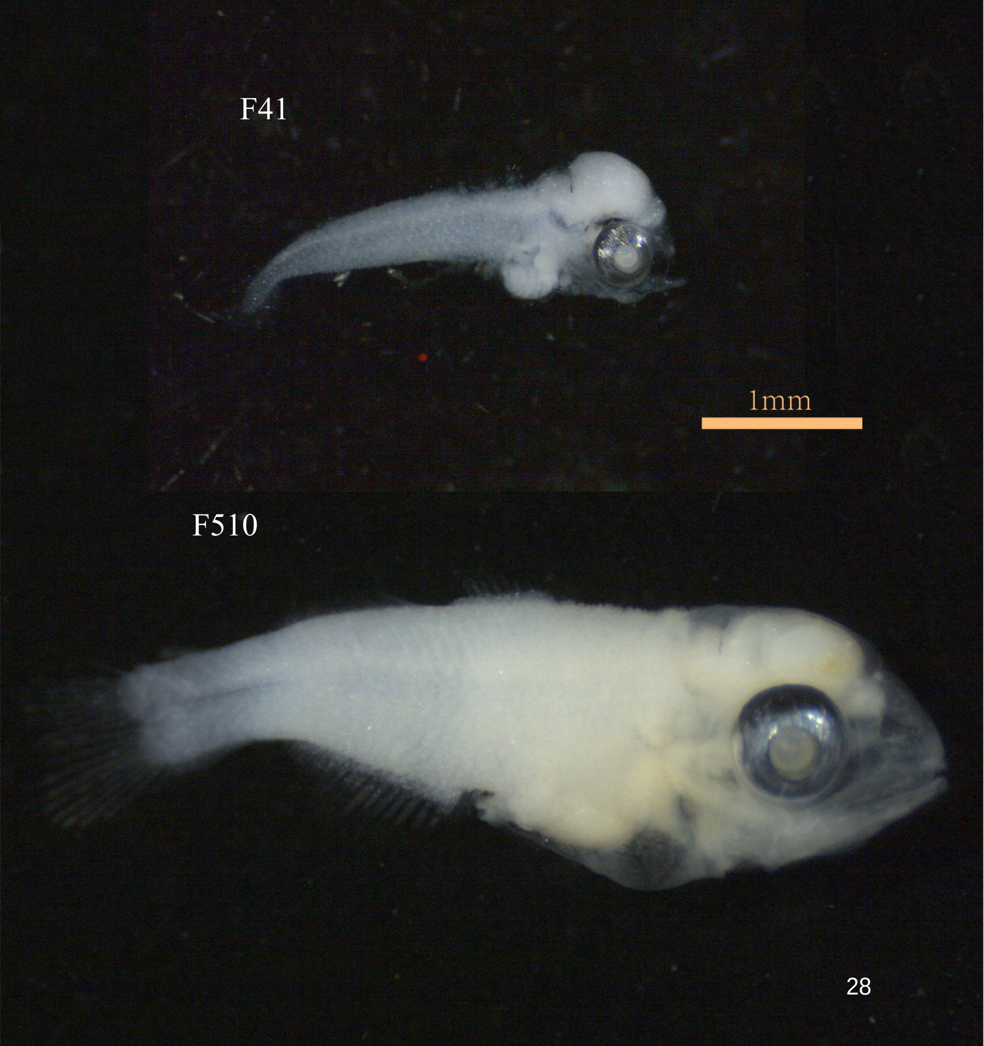
F41
1mm
F510

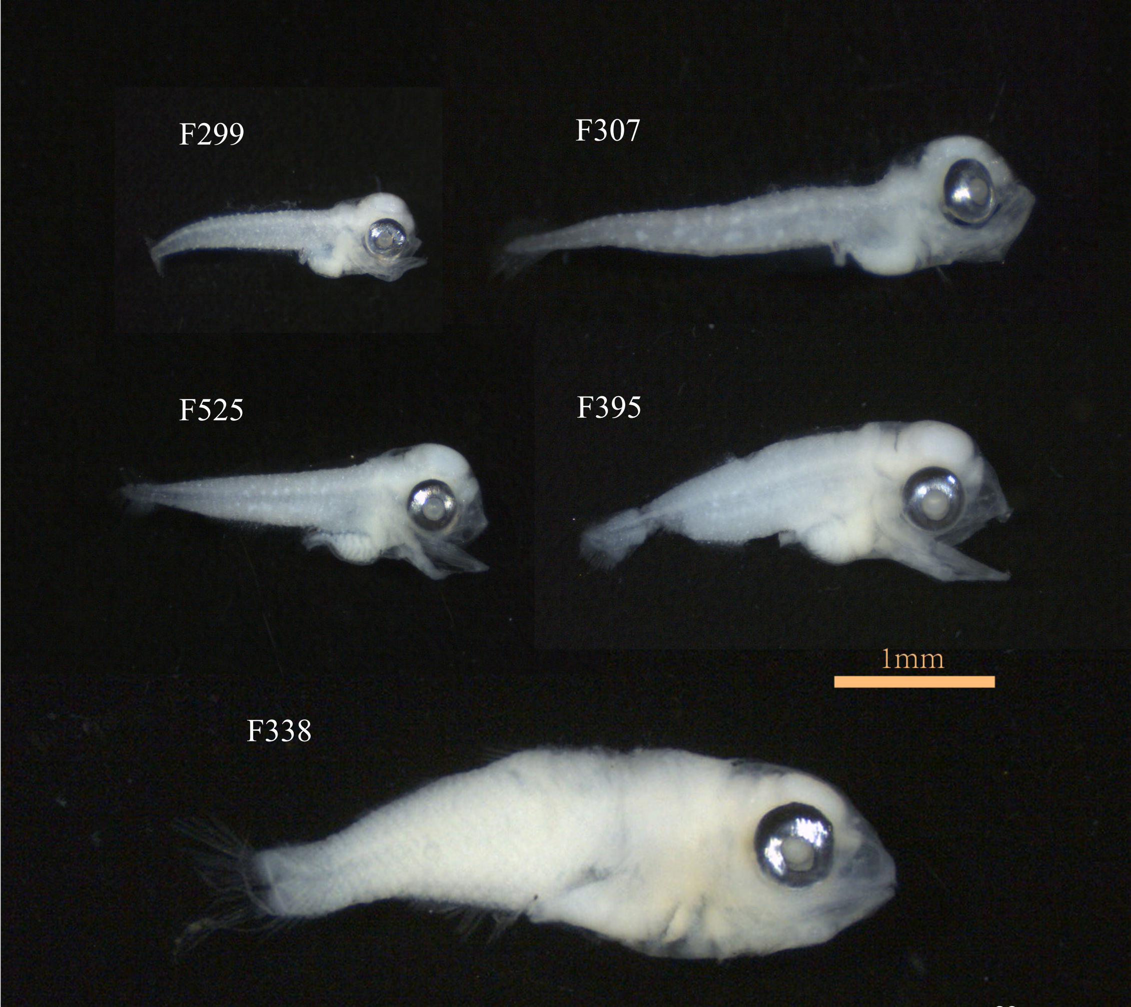
F299
F307
F525
F395
1mm
F338

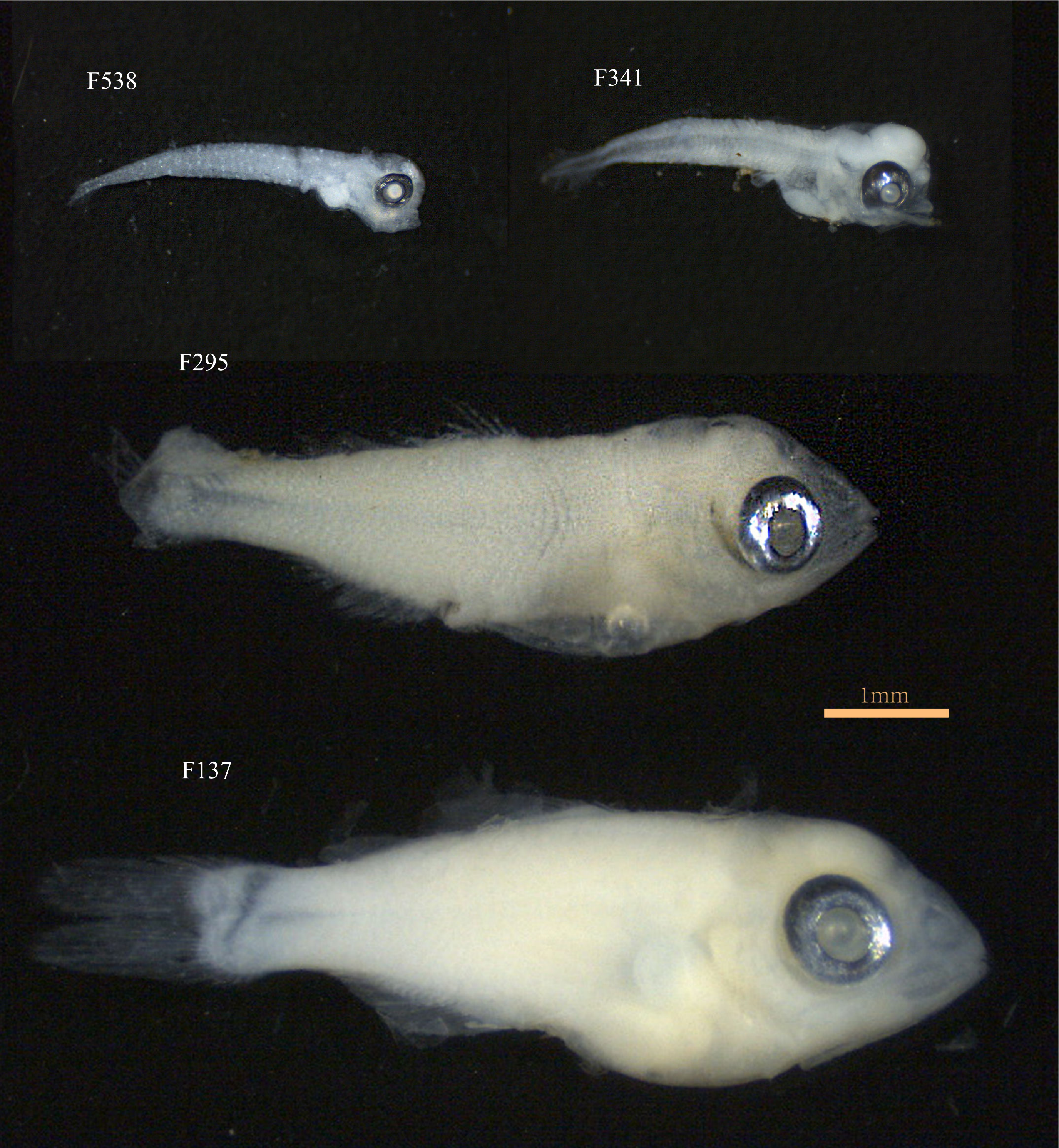
F538
F341
F295
1mm
F137

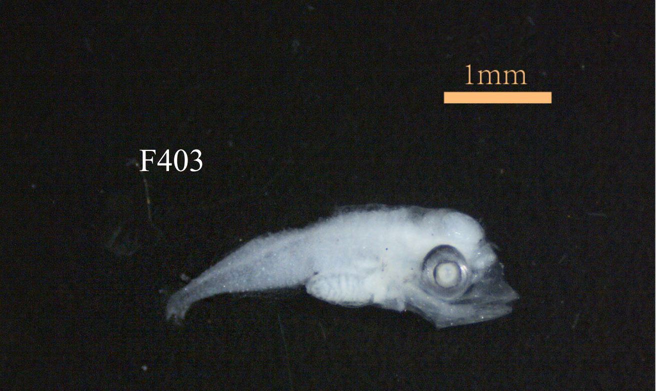
1mm
F403

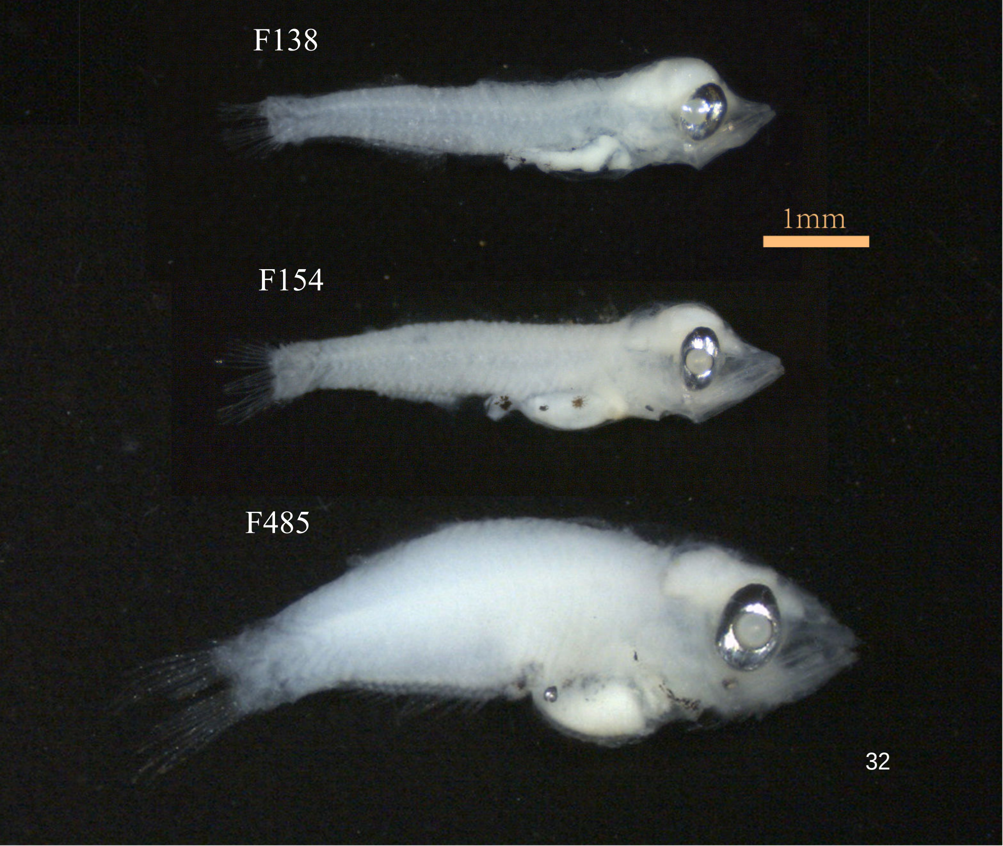
F138
1mm
F154
F485

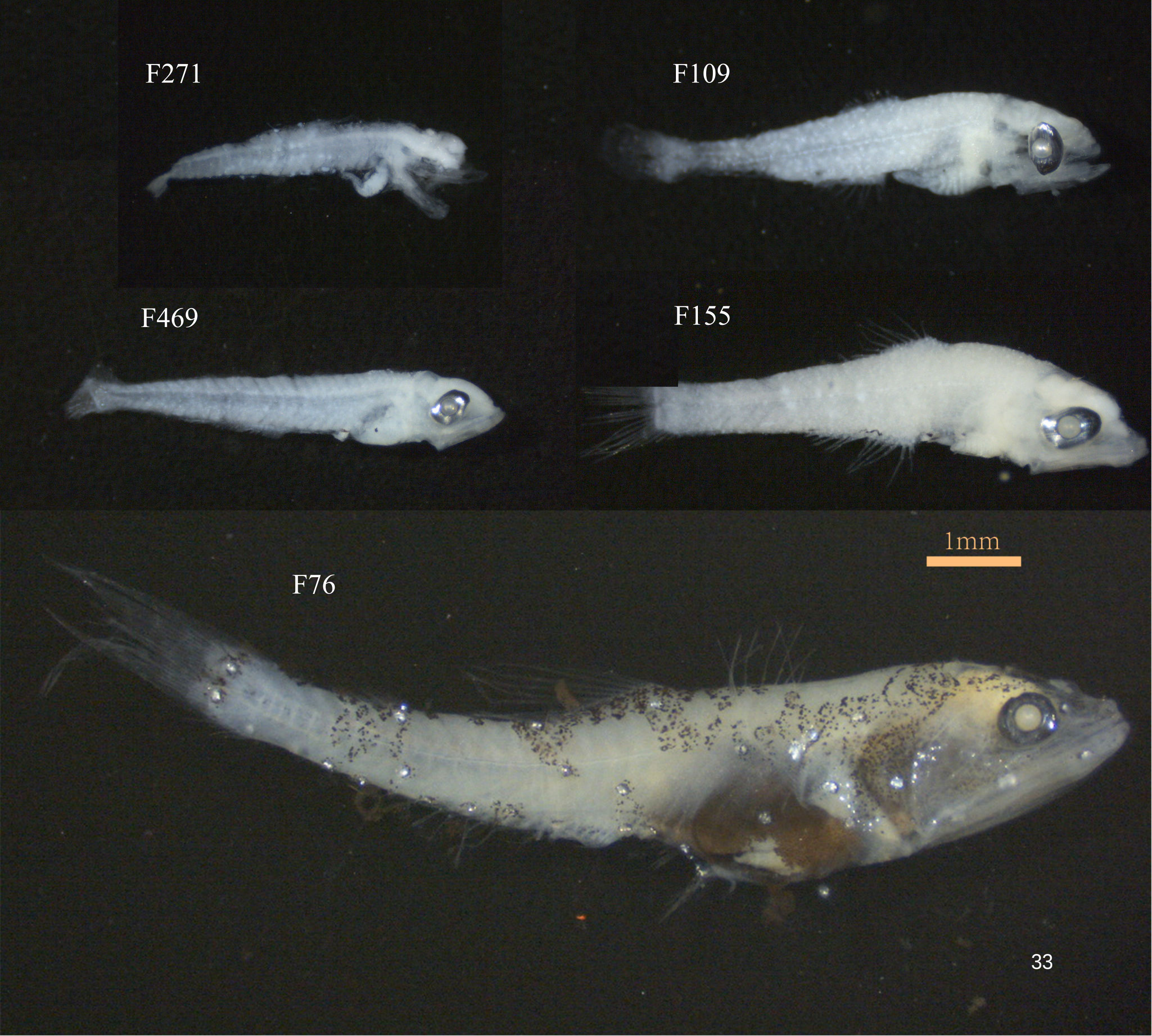
F271
F109
F469
F155
1mm
F76

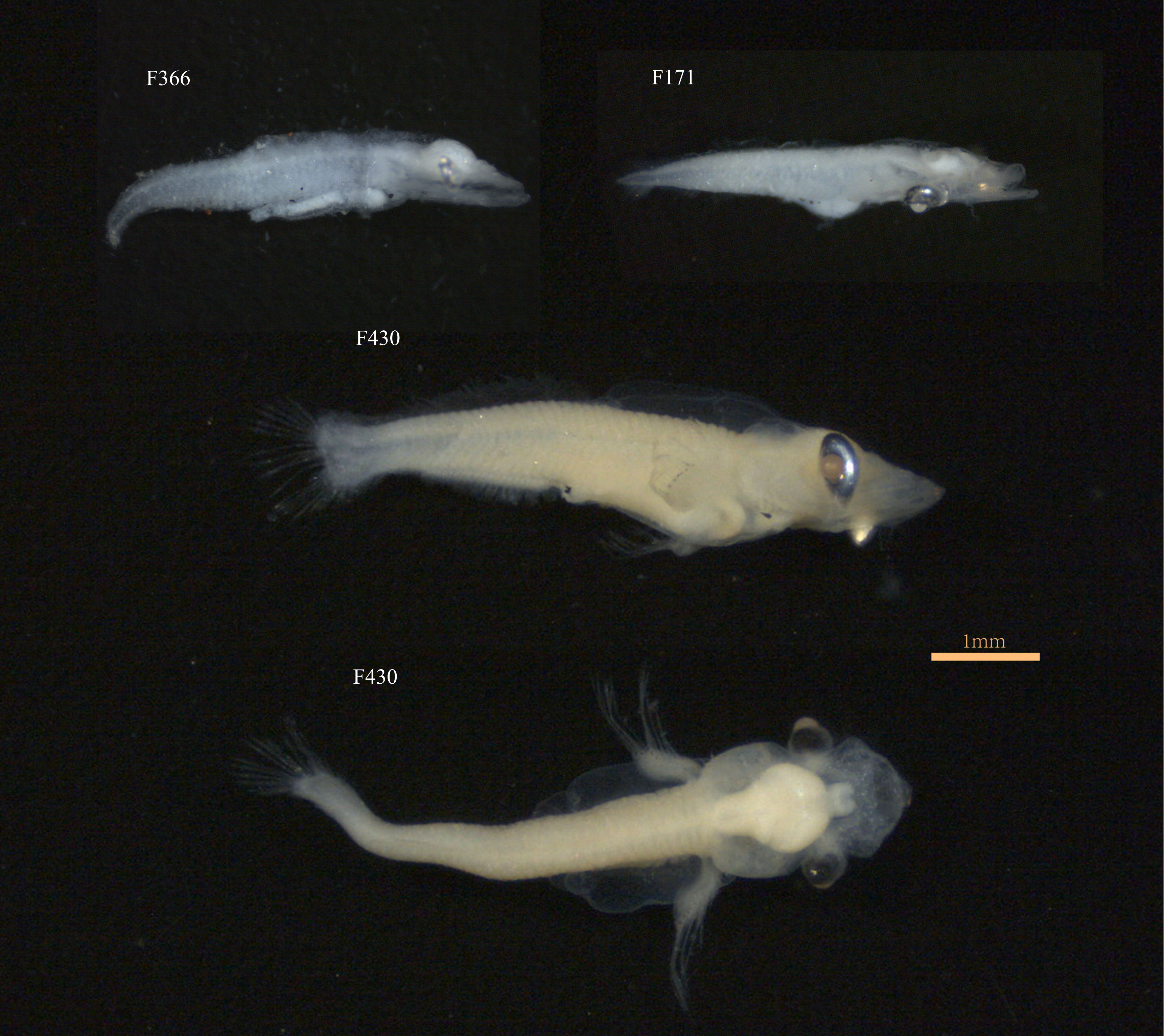
F366
F171
F430
1mm
F430

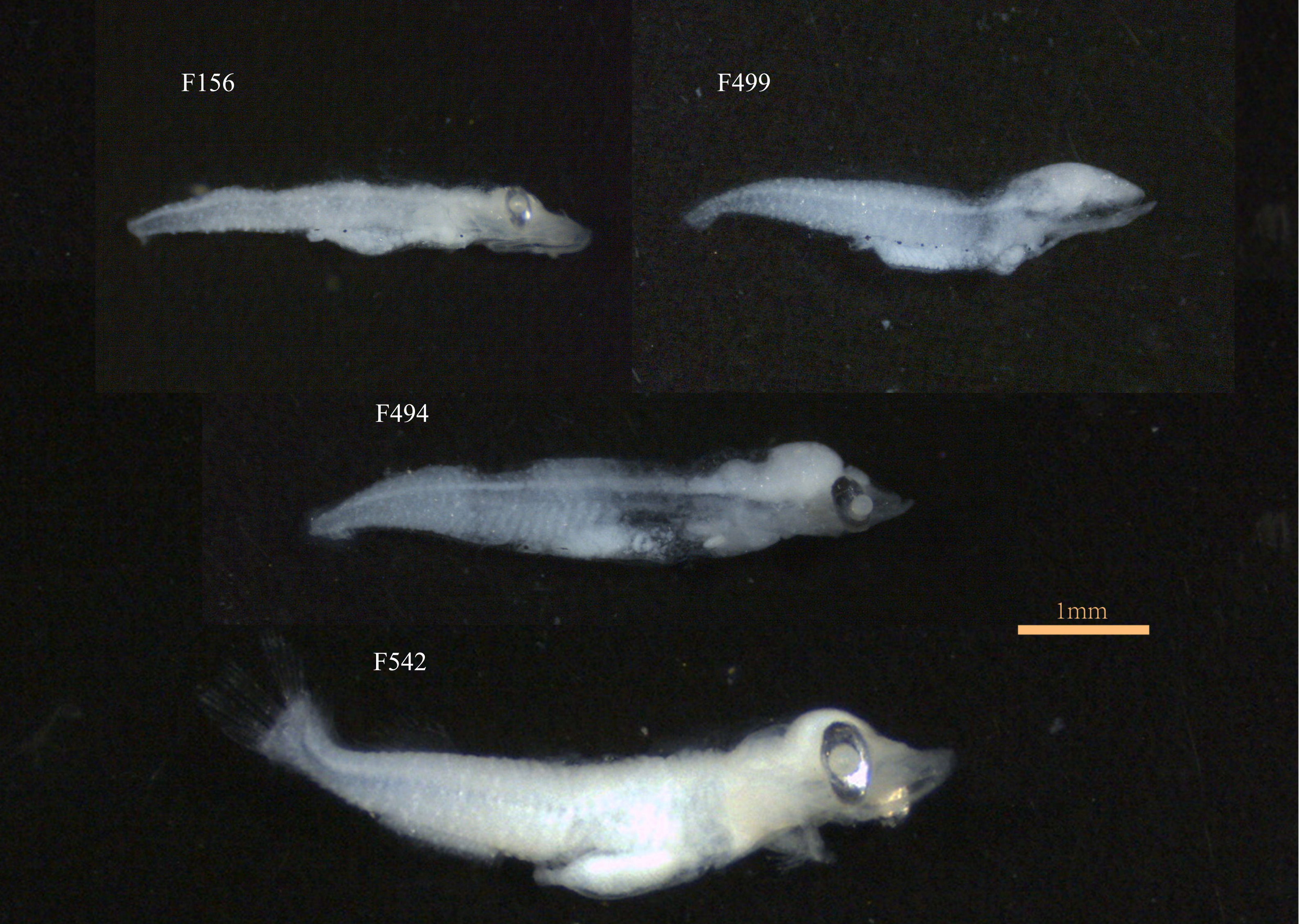
F156
F499
F494
1mm
F542

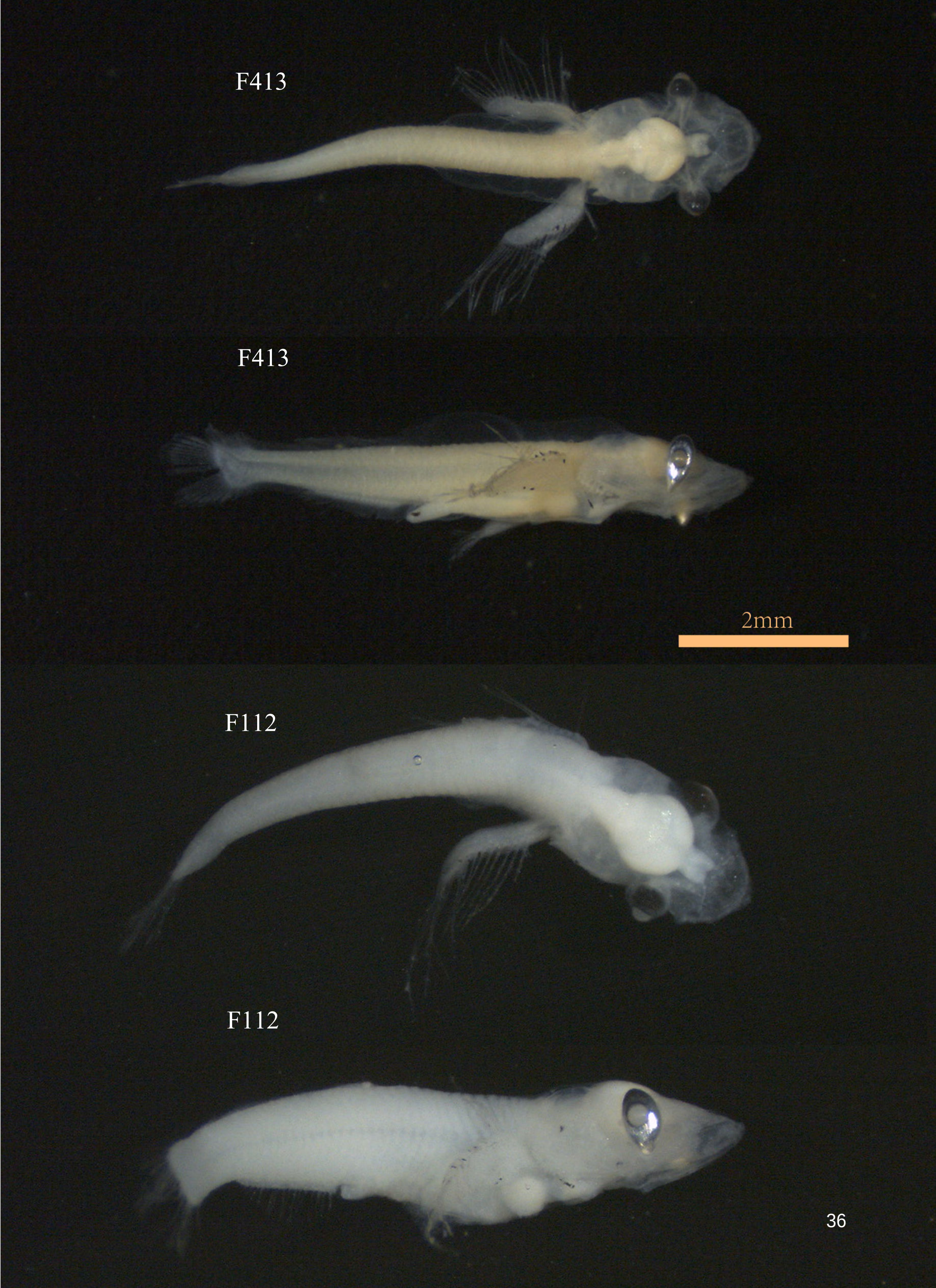
F413
F413
2mm
F112
F112

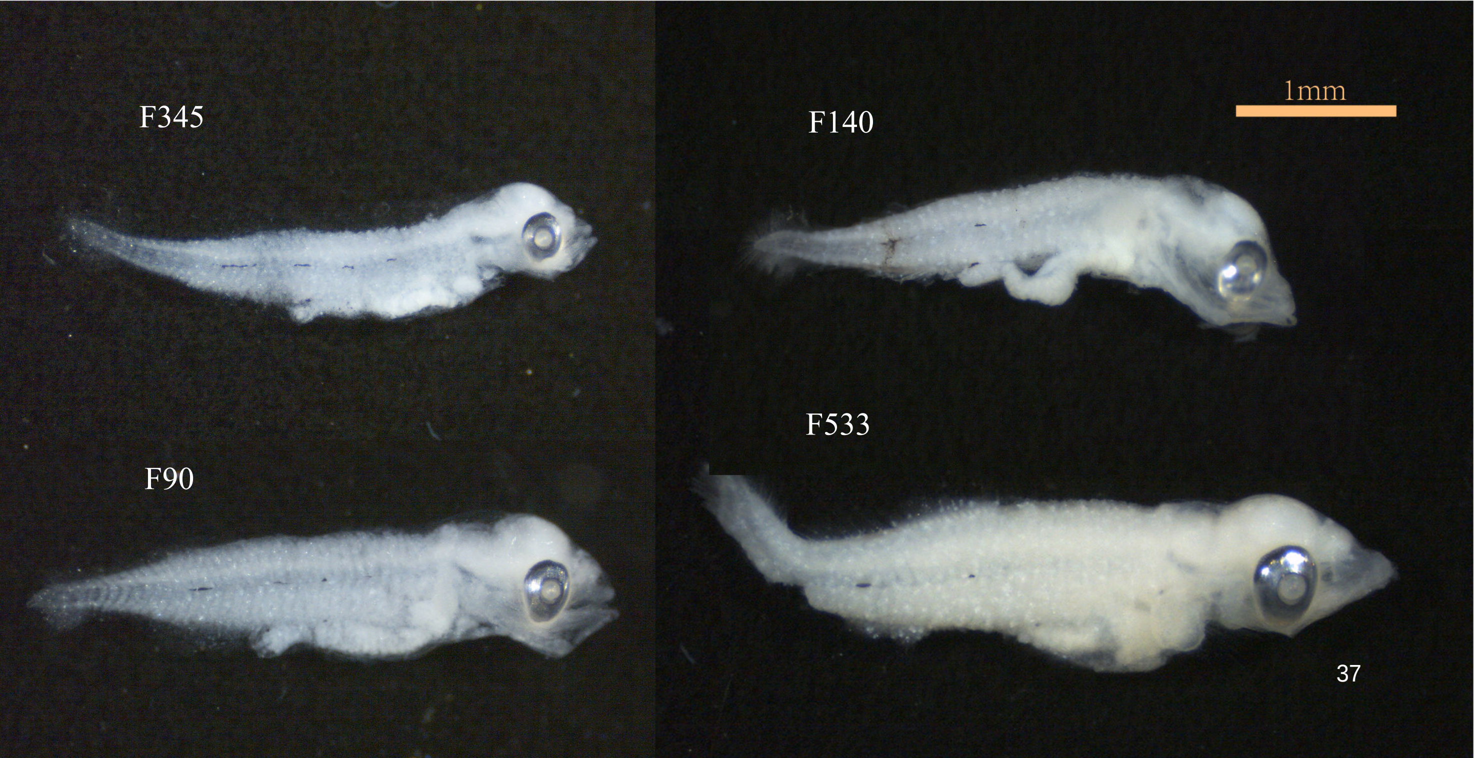
F345
F140
1mm
F533
F90
